# In which sequence? Does it matter?

**DOI:** 10.15694/mep.2020.000098.1

**Published:** 2020-05-13

**Authors:** Manori Amarasekera, Rebecca Anglin

**Affiliations:** 1 School of Medicine

**Keywords:** curriculum review, preclinical, medical education, themes, sequence, pedagogy

## Abstract

This article was migrated. The article was marked as recommended.

A distinctive feature between the delivery of traditional preclinical and clinical curricula is that discipline-based clinical curriculum is delivered in a series of parallel clinical rotations for different groups of students, whereas the curriculum is synchronized across the cohort in preclinical years. Therefore, the sequence of learning themes or topics is more relevant to the preclinical years compared to clinical years in which the beginning and end points of the curriculum are different from one student to another. Many factors, both pedagogical and logistic, influence the optimal sequence of themes for the preclinical curriculum. During the process of reorganizing the sequence of themes for the preclinical curriculum at the University of Notre Dame Australia Fremantle, we identified a relative gap in literature on this topic. Given the importance of the sequence of themes for learning in the preclinical years, we were surprised to learn that publications on this topic are sparse. While sharing the challenges that we came across in our decision making process, we invite the scholars in this area to share their experience. This will undoubtedly benefit curriculum developers and educators in creating or reviewing the preclinical curriculum at their respective institutions.

## Background

Traditionally the medical curricula, whether for undergraduate level or postgraduate level, is presented in two distinctive phases: preclinical and clinical. While there are many schools of thoughts regarding relevance of this distinction in the context of evolving educational theories and practices ( McColl, Bilszta and Harrap, 2012; Prince *et al*., 2005), many medical schools around the world still follow this traditional model of curriculum division. One of the key differences underlying this division is how the curriculum is delivered for learning. In clinical years students follow a discipline-based clinical rotations wherein the learning occurs mostly through clerkships. Discipline-based clinical rotations are organised to ensure that the entire cohort of students get sufficient exposure to clinical teaching and learning opportunities. As such the cohort is divided into small groups of students and each group is allocated a rotation to commence their clinical training. All the groups progress through different rotations until they complete their clinical training. In this setting the arrangement of sequence may not be relevant, however, this is an area for further exploration: if feasible would there be a preferred sequence to facilitate learning in clinical years? Should the order of rotations be tailored to student learning needs or their career goals?

The picture is entirely different in the preclinical years, in which all the students in a cohort start learning a topic or a theme at the same time and progress through the curriculum as a cohort: no random allocation of topics but an identical sequence of topics for all the students. The sequence of themes introduced in preclinical years becomes more relevant because it is the beginning of their learning journey from a relative lack of expertise to being competent for their clinical training. But the question is: what is the best sequence of themes that is most effective for learning in the preclinical years? This was the question that we searched answers for when reviewing the preclinical curriculum of School of Medicine Fremantle (SoMF), University of Notre Dame, Australia.

We did a systematic search using different search strategies on three databases (Ovid MEDLINE, Embase and ERIC) dating back to 1946 and only a handful of papers were found to give some information on rationale for the sequence they have adopted for their preclinical curriculum ( Brooks *et al*., 2015; Klement, Paulsen and Wineski, 2011; Neville and Norman, 2007). This was not quite expected as it is one of the crucial element taken into consideration in designing a preclinical curriculum. The information that is available from medical schools’ websites was limited to an outline of the themes or an introduction to the preclinical curriculum. Various assumptions can be made for the sparsity of open discussions in this area: the sequence or the flow of topics has been successfully established and was not considered to evoke academic interest for ongoing discussions, or has not been included in the publications due to its limited potential in reaching a wider audience compared to focusing on new educational approaches or statistical analysis of student performance in the reviewed curriculum. However, the aim of this article is not to explore the potential reasons for the lack of publications but to invite the educators and curriculum developers to share their untold stories and experiences on this important but under-represented topic in the literature.

Investigation into preclinical or pre-clerkship curriculum of other medical schools through their official websites revealed vast differences in the sequence of themes between them. However, information on the basis or the rationale for adopting a particular sequence was not published. We believe that in spite of thorough consideration has been given to the sequence, these processes has not made known to the scholarly public through publications. For an example, many medical schools place cardiovascular system as one of the early themes in their preclinical curriculum, however, we were unable to identify if this was based on the traditional trend of learning cardiovascular physiology early in a subject-based traditional curriculum or for another reason. Cell structure and function appeared to be the first theme in most medical curricula, while Immunology (which had different titles) varied in its position in the sequence among the schools.

After twelve years of its inception, the preclinical curriculum of SoMF was subjected to a series of curriculum reviews commencing in 2016. As previously published, reviewing body system-based curriculum blocks in an organised manner was given the priority ( Amarasekera, Noakes and Power, 2019). During this process we noticed that both students and the staff have given their feedback on the sequence of themes that they learn particularly in the first year. As Norman (2017) stated that it is not explicit when and why a medical curriculum in a school should be reviewed, however, we felt this is the time to review the sequence of the themes for the preclinical curriculum based on the feedback. Although it sounds like a process that does not require much effort to decide a logical flow to arrange themes, it was far more challenging than we expected. A number of confounding factors embedded in our unique curriculum required thorough analysis before deciding the most effective sequence for learning. The PBL-based curriculum, integration of domains (basic clinical sciences, clinical and communication skills, population and preventative health, professional and personal development, research and aboriginal health), educational theories underpinning approaches to learning at different stages of the curriculum delivery, and differences in experience and knowledge of candidates at entry to the SoMF were among the many factors that influenced the decision making process.

It could have been an easy task to adopt an elegant sequence of themes in a traditional subject-based curriculum as any standard text book would elaborate a logical flow of a particular subject. In an integrated curriculum it is challenging to come to a consensus as basic sciences and clinical skills should evolve together in a sensible flow on its own, yet in a combined theme. For an example, basic science as a discipline would arguably prefer to start introducing to cells and genes while clinical sciences would prefer to introduce body systems and how they interact. The nature of the PBL-based learning that is organised into body systems add another layer of complexity to sort out an efficient and practical sequence. A clinical problem of cervical cancer would have been a good example for a PBL-based learning about cellular changes in cancer. This can be introduced early in the curriculum to facilitate understanding of normal cellular processes and how their deviation leads to pathology. This would also introduce the principles of screening for disease which is foundational knowledge in population and preventative health. However, clinical skills associated with examination of female pelvis would preferably be taught later in the curriculum as it involves sensitive examination and the learner is required to have mastered more comfortable examination procedures prior to this.

## What influenced our sequence?

The medical program at The University of Notre Dame Fremantle, Australia is a postgraduate course and an undergraduate degree in Science or health related field is not a prerequisite to gain entry to the medical program. Graduates with different academic backgrounds and different working experiences commence the PBL-based curriculum at the same time. Students with a science background may find it comfortable to start learning cellular mechanisms at the beginning because their prior knowledge may make them feel more confident. On the other hand, students who have several years of clinical experience in health services, such as nursing and paramedical sciences may find it comfortable to commence the course with more clinically oriented themes. Differences in student preferences may have an impact on how well they are confident and engaged during the first few weeks of the preclinical course. It can be challenging to design a sequence for which pedagogy is the foundation yet capable of accommodating active engagement of learners with different interests and capacities.

Interprofessional education and community engagement placements are increasingly incorporated into medical curricular to enable medical graduates ready to work collaboratively with other health professionals and communities. Many factors which are mostly external logistic factors influence the timing of such activities. This in turn will have an impact on the flow of themes in a sequence. A compromise would be either to change the sequence to accommodate such activities without keeping them disjointed from the PBL case scenarios or to keep the activities as isolated tasks in the curriculum. The latter would maintain the pedagogy of the sequence, however, the themes will be disconnected from the PBL scenarios.

After an exhaustive task of collaboration and communication we identified the best possible sequence of themes for year one of the PBL- based preclinical curriculum. We have implemented the reviewed sequence of themes in the form of curriculum blocks in 2019 without making significant changes to the overall theme for year one, which is learning the ‘normal structure and function of the human body to understand the health and disease’. Table 1 summarises the sequence of themes for year one curriculum, how they are organised in the context of body systems taking the complexity of associated PBL scenarios into account and the rationale for the sequence.

**Table 1.** Sequence of themes organised into curriculum blocks for year one medical curriculum of SoMF, University of Notre Dame, Australia

Curriculum Block	Theme	Summary of concepts learned
Homeostasis	Orgnisation of the human body from biomolecules to whole organism and importance of homeostasis in health	This block provides an introduction to human anatomy, basics in metabolism using carbohydrate metabolism as an example, key concepts in human physiology using cardiovascular system as an example. It also provides the opportunity to identify key elements of professional development (duty of care and code of conduct). Introduction to rural and population health is also embedded in this block. This introductory block prepares the learner to understand the key concepts in medicine.
Genetics	The next level of organisational hierarchy from biomolecules to whole organism: cell biology with a focus on genetics	This block while focusing on cell cycle and regulation, also introduces several body systems: respiratory system through a genetic disease affecting the respiratory tract, haematology through a haemoglobinopathy, gastrointestinal tract/ hepatobiliary system through bilirubin metabolism and abnormal pigments. Impact of disease on an individual and their family and evidence based medicine are also integrated into this block.
Upper Respiratory Tract	Experiential learning: rural immersion placement	Using ear infection and hearing impairment as examples, this block introduces social and cultural aspects of disease and aboriginal health. The timing of this block is mainly influenced by logistical factors of the excursion.
Musculoskeletal	Cells are organised into specialised tissues	This block introduces different types of tissues using musculoskeletal system as an example. Basics of endocrine regulation and muscle physiology are also introduced. Public health measures addressing common and significant health issues is built into this block as foundational knowledge in preventative health.
Body Defence Mechanisms	Interactions between cells, molecules and tissues	Introduction to immunology using inflammation of the skin as an example is the opening theme of this block. Learning is also extended to microbiology using bacterial infections as examples. This block also introduces the concept of pathogenesis of a disease and how this relates to principles of treatment.
Gastrointestinal	Implications of host-pathogen interactions	While students learn anatomy and physiology of the gastrointestinal tract in detail, they extend their learning in immunology through its applications in health and disease. Virology, parasitology and environmental health are learned expanding the discussion on interactions between the host and pathogens.
Cardiovascular	Back to homeostasis: heart as a pump	Revisiting the concept of homeostasis using cardiovascular system in detail is the theme of this block. Lipid metabolism in health and disease is used to further explore disease pathogenesis. This block also highlights the importance of risk assessment and disease prevention as an effective strategy in population health.
Lower Respiratory Tract	Heart and lungs are interrelated in maintaining homeostasis	Anatomy and physiology of respiration, and tobacco related harm are the topics of this block. Students discuss implications of sudden or unexpected death of a patient to the doctor, and the strategies to manage their emotions.
Neurology	Nervous system as the centre of homeostasis	This block provides the foundations of neuroanatomy and neurophysiology. In addition, interprofessional team work, and alcohol related harm and strategies to prevent them are also discussed. Burden of chronic diseases on individuals and health care system is analysed.
Mental Health	‘Homeostasis’ of mind	This block introduces the psychological issues that a person may develop across the lifespan: emotional development in adolescence, depression as a possible mental health issue in middle aged and changes in cognition in the elderly. As an extension of the Neurology block, mental health block provides the opportunity to learn the applications of neuroanatomy and neurophysiology. Key social issues such as domestic violence and ageing population are also discussed. Nutrition for healthy living is integrated into this block.
Urogenital	Kidneys work in conjunction with cardiovascular and respiratory systems to maintain homeostasis	Anatomy and physiology of the renal system to understand the renal regulation of key homeostatic mechanisms (fluid, electrolytes, acid-base) is the core of this block. In addition, it introduces the male urogenital system and provides the opportunity to develop skills in taking a history and performing physical examination on sensitive matters. Screening for disease at population level is discussed in detail.
Reproduction	To where it began: the cell	This block discusses the female reproductive system. It expands to embryogenesis and fetal development. This also provides the platform to discuss some ethical issues such as miscarriage, assisted reproduction technology, medical error and negligence. Revisiting the cell cycle and genetics, neoplasia and metastasis are also discussed. The latter leads to the analysis of prognostic factors and survival curves.
Multi-system review	Perturbations in homeostasis in one system have flow-on effects on other systems	As the last block of the first year curriculum, it provides the opportunity to review the body systems while recognising the anatomical and physiological interrelationship between the systems.

Our experience highlights many challenges that curriculum designers and medical educators may come across in developing the best sequence of themes without compromising the pedagogy of learning. It will be beneficial for enthusiastic curriculum developers and reviewers if similar experiences are published and recommendations are shared to explore the many possibilities and to be aware of the unexpected challenges. Therefore, we expect that our discussion will encourage others to comment on how similar or different their process for deciding the sequence was. This will contribute to fill the gap in literature on this under-represented yet important topic as the sequence of themes matters in the preclinical curriculum!

The difficulty in measuring the success of a new curriculum or changes to the curriculum beyond the level of student satisfaction is well known ( Norman, 2017). However, our process was initiated by the consistent pattern of student and staff feedback, hence we believed, that it was reasonable to return to the audience. After completion of a full cycle of revised year one curriculum we sought the student and staff feedback. It was an overwhelming positive response to the change. While this was encouraging to the school, it also raised the expectation of further changes to the rest of the preclinical curriculum, especially the sequence of themes for the rest of the preclinical curriculum and beyond!

## Take Home Messages

• Sequence of learning themes in preclinical curriculum is important for learning and student engagement.
• Pedagogy and many other factors influence the sequence of themes.
• There is a relative gap in literature for the rationale in adopting a specific sequence of themes for the preclinical curriculum.

## Notes On Contributors

**Manori Amarasekera**, MBBS, MPhil, PhD, Grad Cert HPE, is the Preclinical Curriculum Coordinator, School of Medicine, University of Notre Dame Australia, Fremantle WA, Australia.

**Rebecca Anglin**, MD, PhD, FRCPC, FRANZCP, is the Associate Dean (Preclinical), School of Medicine, University of Notre Dame Australia, Fremantle WA, Australia.
