# Supplementary material for: In which sequence? Does it matter?
Source: MedEdPublish (2016). 2020 May 13;9:98. [Version 1] doi: 10.15694/mep.2020.000098.1 (PMC10697447; doi:10.15694/mep.2020.000098.1)
Supplement: Peer review history

# Reviewer report: Reviewer response for version 1

**DOI:** 10.21956/mep.20077.r30892

**Published:** 2020-10-13

**Authors:** Ken Masters

**Affiliations:** 1 Sultan Qaboos University

This review has been migrated. The reviewer awarded 3 stars out of 5 An interesting paper dealing with the possible implications of the order of various themes in pre-clinical education. As one would expect the sequence to be well designed (especially in light of the concept of the “spiral curriculum”), I was as surprised as the authors to find a paucity of research on the topic.The authors do lay out the sequence of the topics very clearly, and I agree that there would be some value to medical schools’ sharing their sequences, but, in order for this paper to have real value, more information is required, otherwise, the paper threatens to simply be a list of topics taught, supported with a little elaboration. Specifically:• Time on each topic: How much time is spent on each topic? (This includes not only total time, but the time devoted to individual activities).• Methods of teaching: the authors identify their method as PBL, but PBL exists in so many flavours, with a wide variety of expectations regarding supporting materials and activities. I realise that the focus is on the sequence, but a paragraph giving a description of a typical case would help. This is especially important because PBL, by its very nature, is so inclusive of content, and the topics appear to be quite segmented, so, one would wish to see how the segmentation necessitated by the sequence is balanced against the comprehensive nature of PBL.• The impact on the later years - was this evaluated?• The evaluation is tantalisingly given, but with no detail, so the strengths and weaknesses are unknown. I realise that the authors have not submitted this as a research paper, but, if ethics considerations are met, some results would be usefulIt is possible that the frustration I feel at the paper is because the authors have presented a small portion of something that may be part of a much larger project. I feel that I have been given a taste from the chef’s ladle but have been denied the opportunity of eating the full meal. :-(

Reviewer Expertise:

NA

No decision status is available

# Reviewer report: Reviewer response for version 1

**DOI:** 10.21956/mep.20077.r30893

**Published:** 2020-05-14

**Authors:** P Ravi Shankar

**Affiliations:** 1 American International Medical University

This review has been migrated. The reviewer awarded 4 stars out of 5 I enjoyed reading this paper on the preclinical curriculum. The sequence of the curriculum is an important issue and I am glad the authors have brought this out for discussion. I had faced this issue at a medical school in Aruba, Dutch Caribbean when the school shifted from a discipline-based to an integrated organ system-based curriculum. As Chair of the Curriculum Committee I had played an important role in developing and implementing the integrated curriculum. Along with this change there was also an emphasis on small group learning, communication skills, health humanities and other topics. We also introduced early clinical exposure. One of the first questions we had to work out is whether to have the ‘normal’ first and then go on to the ‘abnormal’ or do both together as an integrated system. Eventually we decided to do an integrated system with both normal and abnormal due to a variety of reasons. We felt this would fit in better with the problem-based learning sessions, the early clinical exposure and the pattern of questions in the USMLE. The next problem was to work out the sequence of different organ systems. As the authors mention there was not much literature on the topic. We decided to start with Fundamental Concepts where basic concepts relevant to different disciplines constituting the basic sciences were introduced. I do admit that the Dean of Basic Sciences and the Chair of the Curriculum Committee were both pharmacologists and the subject did influence the sequencing of the blocks. There was lot of productive interactions and debate in both the curriculum committee and the faculty senate. We decided to have Musculoskeletal as the next system. The topic has heavy Anatomy content but the Anatomy faculty were fine with that. In Pharmacology, a knowledge of the Autonomic Nervous system (ANS) is important before drugs acting on other systems are taught and after a lot of debate during which faculty mentioned that the ANS is intricately linked with the central nervous system we decided to have the Nervous system module after the Musculoskeletal system. While it may be challenging to students, a good knowledge of the system helps students considerably with learning subsequent systems. The next system was the Respiratory followed by the Hematopoietic and then the Cardiovascular system. Then we had the Endocrine and Reproductive system and we closed with Infection and Immunity. I agree with the authors that sharing of experience and of practices among educators is important so that we can learn from each other.

Reviewer Expertise:

NA

No decision status is available
